# Dental Caries Preventive Considerations: Awareness of Undergraduate Dental Students

**DOI:** 10.3390/dj8020031

**Published:** 2020-04-01

**Authors:** Hani M. Nassar

**Affiliations:** Department of Restorative Dentistry, Faculty of Dentistry, King Abdulaziz University, P. O. Box 80209, Jeddah 21589, Saudi Arabia; hnassar@kau.edu.sa; Tel.: +966-12-6403443

**Keywords:** diet, preventive dentistry, xylitol, fluoride, CAMBRA

## Abstract

The aim of this study was to assess awareness and knowledge of undergraduate dental students of common caries-related preventive considerations and to highlight these factors in a concise manner to act as a guide for dental practitioners. A sample of 118 undergraduate students at a local government dental school was included. An interactive survey that contains questions related to common preventive strategies against dental caries was presented to the students. The survey contained 22 questions concerning dietary and therapeutic strategies. Students casted their votes using their mobile cellphones. The correct answer for each question was shown to the students, and further discussion was held. Data was collected, and the statistical analysis was conducted using one-sample *z*- and chi-squared tests at 0.05 significance level. The students answered the questions related to oral hygiene practices, xylitol, and the common knowledge regarding fluoride. The questions related to the use of chlorhexidine, dietary factors, and fluoride formulations were answered mostly incorrectly. The students seemed to grasp details of important concepts of flossing, brushing, reducing frequency of sugar exposure, and the use of fluoride products. Still, more emphasis should be given to increase students’ awareness of dietary guidelines for caries prevention, since adequate knowledge of these modalities is paramount for graduating dentists.

## 1. Introduction

Dental caries remains to be one of the most common diseases affecting humans [1,2]. This disease can be defined as “a biofilm-mediated, diet modulated, multifactorial, non-communicable, dynamic disease resulting in net mineral loss of dental hard tissues” [3]. According to the definition, dental caries is greatly modulated by dietary patterns of the individual among other factors. The caries process can be reversed in its early stages without the need for operative procedures [4]. Still, the use of preventive strategies is considered more beneficial and more cost-effective to deal with this disease’s signs and symptoms, since the techniques focusing on surgical intervention rarely change the oral conditions that caused the disease [5,6].

There has been a recent shift in managing dental caries with more focus on prevention and conservative and minimal intervention strategies in the hope of maintaining tooth structure [7]. The reason for this shift is the proven ineffectiveness of current surgical approaches when managing dental caries [1]. Dentists are encouraged to adopt methods for early detection and diagnosis of caries lesions and manage the situation using individualized treatment modalities based on the patient’s caries risk with the focus on behavioral changes and enhancing local protective factors [8].

Undergraduate operative curricula should focus more on presenting the concepts of dental caries dynamics and clinical diagnosis. This should be complemented with highlighting the factors associated with the onset of the disease, as well as the conditions necessary for its prevention and/or reversal. The discussion of these factors should be held at early stages of the dental curriculum, and the focus should be laid on attempting to assess the student’s knowledge frequently to ensure their understating and alignment with practice during clinical sessions. This knowledge and focused clinical practices are very important to a dentist in order to reduce the dental caries incidence, improve the caries risk status of patients, and manage the existing disease at early stages.

A quick search in the PubMed database for “Cariology Education” or “Dental Caries Education” brings a vast number of reports that discuss implementation of cariology and new concepts for teaching dental caries in undergraduate dental curricula worldwide. These approaches include lecture-based modules [9,10], competency-based cariology management programs [11], and curricula developed by regional dental organizations [12,13,14]. It is worth noting that the majority of the mentioned reports emphasized the shift towards prevention, early diagnosis, and conservative approaches of management (e.g., remineralization) when teaching dental caries [10,14,15,16,17,18,19,20,21]. Further, caries risk assessment is among the most important concepts included in these approaches; it is affected by multiple factors, including dietary aspects, oral hygiene practices, bacterial factors, and fluoride exposure [5,22]. A common model for determining caries risk based on these factors is the Caries Management by Risk Assessment (CAMBRA) [5,23]. These concepts must be included in cariology instruction, and students must be assessed for their understanding of the beforementioned concepts to ensure application of preventive and conservative approaches. Hence, the aim of the current study was to assess the knowledge and awareness of undergraduate dental students of common caries-preventive and therapeutic principles. As a secondary objective, common practices in caries prevention and management in light of the CAMBRA model were stated to act as a concise guide for graduating dentists, as well as for public health professionals and dieticians.

## 2. Materials and Methods

This study was approved by the research ethics committee, Faculty of Dentistry, King Abdulaziz University, institutional review board (IRB protocol #157-11-19 / December 08, 2019), and all the participants voluntarily agreed to participate in the study. One hundred and eighteen fifth-year undergraduate dental students (61 males and 57 females) were enrolled into this study. These students were exposed to the concepts of dental caries dynamics, diagnosis, risk assessment, and conservative management in the previous academic year. The survey setting was a regular lecture hall that contained a digital projector and wireless internet connection; the survey was conducted separately for male and female students one day apart due to cultural restrictions within the country of the study. The survey consisted of 22 multiple choice questions dealing with common concepts of caries-preventive and conservative management that were presented one at a time using the projector (Table 1). The students were asked to join the online survey session by scanning a QR code provided on the screen using the *DirectPoll* online tool (http://directpoll.com/; Netcetera, Zurich, Switzerland). The stem of each question was read aloud by the presenter, and a pause was provided to allow students to cast their votes for the answer individually using their cellphones. After the majority of the students would have voted, the percentages for each option would be shown on the projector’s screen, and the correct answer was highlighted. This was supplemented by discussing the rationale behind each answer and the common misconceptions regarding that particular item. The process was repeated for each item, and the students provided their feedback at the end of the session.

The data from both sessions were stored in the cloud on the polling service’s servers. The data were analyzed in light of five themes: caries risk, oral hygiene practices, dietary factors, fluorides, and bacterial factors. The statistical testing was done using a one-sample *z*-test for comparing a proportion with a fixed number to determine the significance of proportion of the students with the correct answer. Also, a chi-squared test was done to determine the proportion of the students with the correct answer in the male and female groups. All the tests were done using SPSS ver. 17 (SPSS Inc., Chicago, Illinois, USA) at 0.05 significance level.

## 3. Results

Table 1 shows statistical comparisons for each question. Overall, the students correctly answered the questions related to oral hygiene practices, including brushing and flossing. These questions showed more female students answering correctly, except for the recommended duration of brushing, which was not significantly different between male and female students.

Regarding the use of chlorhexidine, the students provided correct answers to all the three questions, even though more correct answers were given by female students. A similar trend was observed when the students were asked about the dietary control with low proportions of students answering correctly the majority of questions. This group of questions was presented to depict their understanding of the common dietary parameters affecting the caries process (Table 2). However, a very high percentage of students recognized xylitol as the most anticariogenic sugar substitute and frequency as the main consideration for sugar consumption in relation to dental caries.

The students were asked four questions concerning fluorides; two questions were answered correctly by the majority of students; however, the students did not provide a lot of correct answers to the questions related to the fluoride concentration in oral hygiene products.

**Figure 1:**
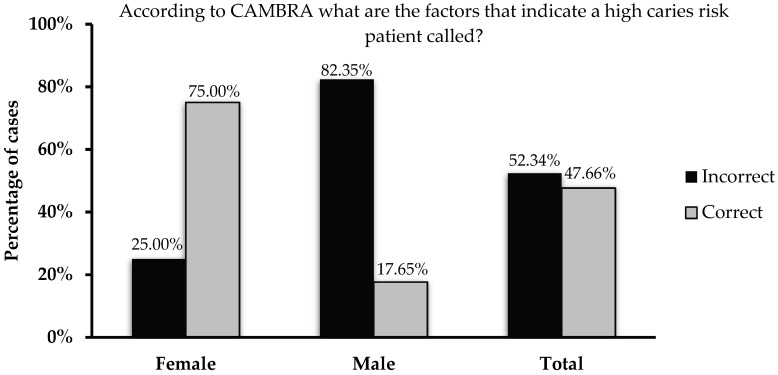
Percentages of female, male, and total students who answered the question regarding the CAMBRA protocol.

Overall, the female students provided more correct answers compared to the male students to the majority of questions. This can be clearly seen in Table 1 that shows more female students providing correct answers as compared to the males. This is very clear in the question related to the disease indicators of the caries management by risk assessment (CAMBRA) model (Figure 1). Table 3 states the recommendations based on this model for managing patients. Still, incorrect answers to question 16 that tested their knowledge regarding cariogenicity of breast and cow milk were predominantly provided by female students.

## 4. Discussion

Dental caries takes a major toll on health services owing to the restorative burden required to replace the diseased tooth structure [6]. Failure to address pathological factors within the oral environment can cause a restored tooth to enter what is known as the “restorative cycle,” since the restoration will not stop the active disease [24]. This cycle means that the restored tooth is subjected to more extensive and aggressive restorative treatments that are frequently replaced by another until the tooth is considered unrestorable owing to little remaining tooth structure. It is estimated that two thirds of restorative treatments are performed on previously restored teeth [25]. Despite this, dental caries lesions can be predictably prevented if early strategies are implemented [1,6]. These include oral hygiene practices, dietary control, fluoride therapy, antimicrobials, sealants, and glass ionomer restorations.

The primary etiology of dental caries diseases is the disturbance in the balance between protective and pathological factors within the oral cavity in favor of the growth of cariogenic bacteria [26]. If these microorganisms continue to thrive by fermenting dietary carbohydrates, they produce lactic acid that can demineralize the hydroxyapatite crystals within the tooth structure. Usually, this mineral loss could be regained in the presence of fluorides and the supersaturation status of saliva. However, if these protective factors are compromised, such as in cases with reduced salivary flow, lack of fluorides, or increased exposure to fermentable carbohydrates, continuous demineralization takes place, tipping the demineralization/remineralization balance and leading to the development of caries lesions. Still, at early stages, these lesions can be remineralized if protective factors are enhanced; however, if the acidic challenges are too extreme or persist for prolonged periods of time, irreversible cavitation can take place, necessitating surgical intervention to remove the diseased tissue according to the conservative management strategies to prevent reoccurrence of the disease [5,27].

The use of fluorides is considered one of the most effective strategies to decrease dental caries incidence and to help remineralize early lesions [4,28]. Among the many forms of fluoride delivery, toothpaste is considered effective in maintaining adequate fluoride levels for caries prevention and management [29]. Knowledge of the amounts and concentrations available in common dental products is crucial in providing the patient with a proper treatment plan and to avoid complications. The students in the current investigation do not seem to be aware of the different concentrations found in common dental products. Further, they are not aware of the major disadvantage of its use, which is toxicity. Based on these findings, additional theory sessions should be considered in order to expose students to the basic mechanisms of action of fluorides to enhance mineral uptake by the tooth structure and to discuss possible side effects due to overexposure to fluorides. The principle modes of fluoride action include: 1) increasing remineralization by enhancing the uptake of calcium and phosphates, 2) decreasing demineralization by the formation of fluorohydroxyapatite crystals that are larger and more resistant to acidic challenges, and 3) inhibiting bacterial enzymes, such as enolase [30,31].

Reducing the dental biofilm (formerly known as dental plaque) can aid in reducing the magnitude of acidic challenges and their frequency. This can be achieved via a combination of physical and pharmacological strategies. Tooth brushing and flossing are recommended to patients to clean teeth surfaces in order to reduce the bacterial load and subsequent demineralization. Brushing is recommended twice daily for 2 minutes using a soft toothbrush accompanied by a fluoride-containing toothpaste [32]. Further, the abrasive potential of toothpastes is also very important to be considered, and highly abrasive formulations, such as whitening dentifrices, should be avoided in cases with incipient lesions [33,34]. Although dental flossing can reduce the amounts of biofilm interproximally, no direct correlation between flossing and reducing proximal caries lesions was established. In addition to the small role of xylitol as an agent to decrease *Streptococcus mutans* load, chlorhexidine is considered very effective in patients with a high caries risk with an increased *S. mutans* load [35,36,37]. It should be mentioned that chlorhexidine is not very effective against *Lactobacilli*, which are more sensitive to dietary modifications [37]. In the present investigation, more than half of the cohort recognized this.

The increase in sugar consumption is linked to the increased incidence of dental caries in the literature [22]. Simple sugars can cause more dental caries owing to their faster metabolization by cariogenic bacteria [38]. Of course, different dietary habits can contribute to the dental caries process differently. Table 2 summarizes some dietary parameters and the effect on the caries process. Frequency is one of the major factors affecting the caries balance [22,39]. A more frequent exposure to fermentable carbohydrates leads to more demineralization episodes leaving the saliva struggling to neutralize these acidic challenges [40]. This factor was predominantly identified by the students. A related factor is the duration of sugar consumption; prolonged periods of sugar consumption lead to sustained lactic acid production by cariogenic bacteria causing the process of demineralization to remain for as long as the sugars are being consumed. High frequency and long duration of sugar consumption are two of the factors that will cause the plaque and salivary pH level to decrease providing a suitable acidic environment for dental caries to develop [39]. Overall, patients should be instructed to reduce the frequency and duration of fermentable carbohydrates consumption. This should be accompanied by substitution with healthy snacks and protective foods, such as nuts and cheese.

Xylitol is considered one of the bulk sweeteners or sugar alcohols [41], and by far is considered the one with the most anticariogenic potential [42] with multiple studies showing its ability to reduce the *Streptococcus mutans* load in plaque and saliva [43,44,45,46]. Utilizing a regimen containing a xylitol chewing gum is recommended when caries risk assessment models are considered [22,23]; for an overview, please refer to the recent review by Nassar [47]. The students seem to grasp this concept very well, and the majority of them (approximately 92%) recognized that xylitol is the only sugar substitute with an anticariogenic potential (Table 1).

The Caries Management by Risk Assessment (CAMBRA) model is one of the well-established caries risk assessment models utilized to depict a patient’s caries risk. It categorizes patients into four categories based on certain parameters. The presence of signs of the disease (also known as disease indicators, which the knowledge among the students in this cohort was less than ideal, Figure 1) establishes the patient as being at high risk of developing new caries lesions. This can be complicated by a reduced salivary flow rate below normal levels (stimulated flow rate below 1.0 mL/min.) lowering the remineralization power of the saliva and providing better conditions for an unbuffered acidic environment. This consequently places the individual in the extremely high caries risk category. If disease indicators are not present, a comparative evaluation of protective and pathological factors should be considered. The presence of more pathological factors places the patient in the moderate caries risk category; and on the contrary, more protective factors indicate a low caries risk status [22].

After determining the patient’s caries risk, the CAMBRA guidelines can provide some management and preventive regimens that can be further tailored based on the case (Table 3). The CAMBRA model recommends a combination of the strategies described above which can be tailored according to the need. Additional methods that were not discussed include calcium phosphate products, neutralizing agents, and saliva substitutes (for details, please refer to the revised CAMBRA guidelines [48]). Of course, the CAMBRA model also promotes detection of caries lesions as early as possible in order to maximize the benefits of early conservative management strategies.

## 5. Conclusions

It is of utmost importance to educate the new generation of dentists to be able to distinguish the criteria causing the increased risk of dental caries. This would allow the formulation of individualized prevention (and management) plans that would allow patients to keep their teeth healthy for long periods and to reduce the need of restorative procedures. Also, it would be advantageous that dieticians and other professionals working in the dietary and health-related professions become familiar with dietary patterns and caries-inducing factors in order to reduce the prevalence of the dental caries disease worldwide, since it remains a global public health issue. Based on the findings of this investigation, emphasis on the concepts of caries risk assessment, especially on dietary factors and fluoride formulations, should be considered in undergraduate dental curricula.

Speaking of limitations of the present study, undergraduate dental students require more exposure to caries preventive and management strategies. This should be complemented by frequent emphasis on the clinical application of these modalities during regular educational encounters and sessions. Restriction of dietary fermentable carbohydrates, exposure to fluoride-containing products, and early caries diagnosis should be emphasized in order to prevent dental caries and/or reduce the need of invasive restorative treatments.

## Figures and Tables

**Table 1:** The questions included in the survey with the correct answer in brackets (actual survey questions were not dichotomous), as well as statistical comparisons for each question of the survey showing proportions of correct vs. incorrect answers and proportions of female (n = 57) vs. male (n = 61) students with correct answers (total n = 118).

Question [Correct Answer]	Proportion of Answers ^1^		Proportion of the Students with the Correct Answer ^2^	
	Correct (%)	Incorrect (%)	Females (%)	Males (%)
Disease indicators are the factors that indicate high caries risk, according to the CAMBRA (caries management by risk assessment) model [yes]	47.7	52.3	**75.0**	**17.6**
Proximal caries lesions can be reduced by flossing [no]	**91.6**	**8.4**	**100.0**	**82.7**
Occlusal caries lesions can be reduced by brushing [yes]	**76.6**	**23.4**	**94.4**	**59.6**
The recommended duration of brushing is 2 minutes [yes]	**90.3**	**9.7**	96.4	84.2
The concentration of chlorhexidine prescribed for patients with high risk is 0.12% [yes]	**39.4**	**60.6**	42.9	35.8
Chlorhexidine mouthwash is indicated for patients with at least high caries risk [yes]	57.7	42.3	**69.2**	**47.5**
Taste alteration is among the side effects of chlorhexidine if used for more than 1 week [yes]	**26.2**	**73.8**	**39.2**	**14.3**
Consistency is the main difference between a rinse and a varnish in application [yes]	**63.1**	**36.9**	**76.5**	**51.7**
Sucrose is the most cariogenic disaccharide [yes]	51.3	48.7	53.8	49.2
Lactose is the least cariogenic disaccharide [yes]	**15.1**	**84.9**	14.0	16.1
Simple sugars are less cariogenic than starch [no]	54.9	45.1	57.4	52.5
Substitution method is effective for dietary control [yes]	**15.1**	**84.9**	7.8	21.8
Xylitol is the only bulk sweetener with the anticariogenic potential [yes]	**92.0**	**8.0**	92.5	91.5
Frequency is the most important parameter when sugar consumption is considered [yes]	**72.0**	**28.0**	72.0	71.9
Cheese is among the foods recommended to end the meal with [yes]	40.8	59.2	**64.0**	**18.9**
Breast milk is less cariogenic than cow milk [no]	**30.1**	**69.9**	**11.1**	**47.5**
Toxicity is the main disadvantage of fluorides [yes]	**75.7**	**24.3**	75.0	76.5
A fluoride varnish is recommended to manage active white spot lesions [yes]	**68.2**	**31.8**	**80.8**	**56.4**
The concentration of fluorides in over-the-counter toothpastes is around 1,450 parts-per-million (ppm) [yes]	51.0	49.0	59.6	42.3
The concentration of fluorides in the fluoride varnish is around 22,500 parts-per-million (ppm) [yes]	52.4	47.6	56.9	48.1
Overall, glass ionomer sealants are more recommended than resin sealants [no]	47.6	52.4	40.7	54.9
Dehydration is among the reasons for reduced salivary flow rate [yes]	**3.1**	**96.9**	2.2	3.9

^1^ Bold and underlined values indicate a significant difference between proportions with correct and incorrect answers (*p* < 0.05) for the chi-squared test. ^2^ Bold and underlined values indicate a significant difference between proportions of females and males with correct answers (*p* < 0.05) for the one-sample *z*-test for comparing a proportion with a fixed number.

**Table 2:** Dietary parameters that can affect the caries risk status.

Dietary Parameter of Fermentable Carbohydrates	Effect on Dental Caries Risk
Type:	Simple sugars and fermentable carbohydrates (especially sucrose) have more potential to cause acidic challenges. Breast milk is more cariogenic than cow milk due to lower amounts of minerals and high sugar content.
Frequency:	Increased number of episodes of acidic challenges demanding a wider salivary buffering capability.
Consistency:	Sticky food remains on tooth structure for prolonged periods of time leading to more demineralization and sustained acidic challenges that require morebuffering action. Sweetened liquids can be more easily washed away and are considered less cariogenic than sticky foods.
Amount:	Has little effect if other factors are not considerably high.
Duration:	Increased amount of time that oral pH is below critical pH levels leading to more demineralization.
Sequence:	Ending the meal with a protective food (e.g., cheese or nuts) will reduce the cariogenic potential of the meal. Nuts provide mechanical cleaning of teeth surfaces. Cheese can help neutralize acids and provides a source of calcium and phosphates.
Pattern:	Frequent snacking on sugar-containing foods increases the caries risk by increasing the frequency of acidic challenges and demanding a more buffering action. Furthermore, combining a highly cariogenic liquid with a sticky food with low cariogenicity enhances the cariogenic potential of the liquid.

**Table 3:** Caries risk assessment and management guidelines based on the CAMBRA risk levels (adapted from Jenson et al., 2007).

Risk Status	Frequency of Bitewing Radiographs ^1^	Frequency of Caries Recall Exams	At-Home Fluorides	In-Office Fluorides	Antimicrobial Therapy	Saliva Substitutes	Calcium Phosphate Products	Sealants
Low risk	Every 24–36 months	Every 6–12 months	OTC ^2^ fluoride- containing toothpaste twice daily	Not needed	CHX ^3^: not needed Xylitol ^4^: not needed	Not needed	Not needed	Optional
Moderate risk	Every 18–24 months	Every 4–6 months	OTC fluoride- containing toothpaste twice daily 0.05% sodium fluoride (NaF) rinse daily	Not needed	CHX: not needed Xylitol: yes	Not needed	Not needed	Optional
High risk	Every 6–18 months	Every 3–4 months	1.1% NaF toothpaste twice daily	1–3 applications of fluoride varnish	CHX: yes Xylitol: yes	Not needed	Optional	Yes
Extremely high risk	Every 6 months	Every 3 months	1.1% NaF toothpaste twice daily 0.05% NaF rinse after snacking, breakfast, and lunch	1–3 applications of fluoride varnish	CHX: yes Xylitol: yes	Rinses as needed if mouth feels dry, after snacking, bedtime, and after breakfast	Calcium/ phosphate paste twice daily	Yes

^1^ The frequency of radiographs and re-care visits takes into consideration the access to care and patient’s compliance. ^2^ OTC: over-the-counter toothpaste. ^3^ Two tabs of gum or two candies four times daily corresponding to 6–10 grams/day. ^4^ CHX: 0.12% chlorhexidine, 10 mL of rinse for one minute daily for one week each month for 3 months followed by reassessment.
